# Luminal Plasma Treatment for Small Diameter Polyvinyl Alcohol Tubular Scaffolds

**DOI:** 10.3389/fbioe.2019.00117

**Published:** 2019-05-22

**Authors:** Grace Pohan, Pascale Chevallier, Deirdre E. J. Anderson, John W. Tse, Yuan Yao, Matthew W. Hagen, Diego Mantovani, Monica T. Hinds, Evelyn K. F. Yim

**Affiliations:** ^1^Department of Chemical Engineering, University of Waterloo, Waterloo, ON, Canada; ^2^Laboratory for Biomaterials and Bioengineering, CRC-I, Department of Mining, Metallurgical and Materials Engineering, CHU de Québec Research Center, Regenerative Medicine, Laval University, Québec City, QC, Canada; ^3^Department of Biomedical Engineering, Oregon Health & Science University, Portland, OR, United States

**Keywords:** ammonia, radio-frequency, stability, hydrogel, endothelialization

## Abstract

Plasma-based surface modification is recognized as an effective way to activate biomaterial surfaces, and modulate their interactions with cells, extracellular matrix proteins, and other materials. However, treatment of a luminal surface of a tubular scaffold remains non-trivial to perform in small diameter tubes. Polyvinyl alcohol (PVA) hydrogel, which has been widely used for medical applications, lacks functional groups to mediate cell attachment. This poses an issue for vascular applications, as endothelialization in a vascular graft lumen is crucial to maintain long term graft patency. In this study, a Radio Frequency Glow Discharges (RFGD) treatment in the presence of NH_3_ was used to modify the luminal surface of 3-mm diameter dehydrated PVA vascular grafts. The grafted nitrogen containing functional groups demonstrated stability, and *in vitro* endothelialization was successfully maintained for at least 30 days. The plasma-modified PVA displayed a higher percentage of carbonyl groups over the untreated PVA control. Plasma treatment on PVA patterned with microtopographies was also studied, with only the concave microlenses topography demonstrating a significant increase in platelet adhesion. Thus, the study has shown the possibility of modifying a small diameter hydrogel tubular scaffold with the RFGD plasma treatment technique and demonstrated stability in ambient storage conditions for up to 30 days.

## Introduction

Plasma treatment is a well-known technique for activating/functionalizing biomaterial surfaces. The exposure of these surfaces to ionized gas leads to incorporation of chemical species, which results in surface functionalization. For example, it was previously reported that the wettability of plasma treated surfaces improves after the treatment to favor either bonding of coating material or cell adhesion (Nakagawa et al., 2006; Jacobs et al., 2012; Ino et al., 2013; Liu et al., 2014; Cutiongco et al., 2016a; Recek et al., 2016; Jurney et al., 2018). Depending on the gas type and the material that is interacting with the plasma, one can introduce different surface functional groups on the biomaterial surface. For example, silanol (polar) group is formed when treating polydimethyl siloxane (PDMS) with oxygen plasma (Tan et al., 2010), while carboxyl and thiol functional groups can be created by treating the material surface with CO_2_ (Manakhov et al., 2018), and hydrogen sulfide plasma (Thiry et al., 2014), respectively. Moreover, H_2_/N_2_ plasma treatment demonstrated the capability to surface functionalize PVA via the introduction of amide, carboxylic acid, and OH/NH function groups (Ino et al., 2013).

Cold plasma can be generated at low temperature and pressure, and at a high voltage. Such plasma can be produced using Radio Frequency Glow Discharges (RFGD) system, which has been reported for surfaces of tubular, bar, or fibers (Blackman et al., 1994; Wade et al., 2000; Grace and Gerenser, 2003; Cao et al., 2007). To date, only few studies have investigated the effects of plasma treatment on the luminal surface of a tube with diameter smaller or equal to 3 mm (Lin and Cooper, 1995; Kaibara et al., 2000; Cao et al., 2007; Qiu et al., 2017). Kaibara et al. utilized ion coater to modify the inner surface of porous polyurethane tubes, while Qiu et al. utilized Plasma Technology Systems' low pressure plasma reactor (Kaibara et al., 2000; Qiu et al., 2017). However, both methods would require porous tubes to be successful. In contrast, Matsuzawa and Yasuda utilized a semi-continuous RFGD system and pressure difference between inner and outer tube surface to treat inner surfaces of plastic tubes (Matsuzawa and Yasuda, 1984). However, this method is not ideal for treating polymers with a certain degree of porosity. A few other groups used the back-and-forth movement of an electrode outside a plasma discharge chamber to generate uniform plasma along their tube samples (Hatada et al., 1982; Lin and Cooper, 1995; Tseng and Edelman, 1998; Cao et al., 2007). While this method successfully plasma treated tubes, Mantovani et al. reported a similar yet improved approach to perform luminal plasma treatment, as it was shown to have a greater symmetry due to the placement and the geometry of the electrodes (Mantovani et al., 1999).

Polyvinyl alcohol (PVA) is a hydrogel material rich in –OH function group and can be produced from base hydrolization of a polyvinyl acetate ester function group. It has been used in many biomedical applications (Yang et al., 1981; Wan et al., 2002; Kobayashi et al., 2005; Nugent and Higginbotham, 2007; Chaouat et al., 2008; Ding et al., 2012; Fathi et al., 2013; Cutiongco et al., 2015, 2016b) and is non-toxic, non-immunogenic, non-carcinogenic, and highly biocompatible. Importantly, PVA is non-thrombogenic as shown previously (Cutiongco et al., 2015; Anderson et al., 2019). PVA is a good material for making vascular graft as its mechanical property can be tailored to match native blood vessels. Its Young's modulus can range from 0.7 MPa (Wan et al., 2014) to 46 GPa (Peijs et al., 1995) depending on the crosslinking method and the degree of crosslinking between the polymer chains. In addition, a previous study has revealed that PVA has a suitable burst pressure and suture retention strength to withstand pulsatile flow *in vivo* (Chaouat et al., 2008). Studies have also proposed the use of PVA for making small diameter vascular grafts with diameter of < 6 mm using a simple dip casting method of a cylindrical mold (Cutiongco et al., 2016a,b). Hence, PVA is a promising material for providing an off-the shelf synthetic small diameter vascular graft. The performance of small diameter vascular grafts to date has remained disappointing with high restenosis/failure rate (Tordoir et al., 1995; Tan et al., 2011). Fabricating an off-the-shelf vascular grafts can, therefore, be one of the ways to fulfill the unmet medical need.

While PVA can be fabricated into small diameter tubular graft with suitable mechanical and material properties as vascular graft, the hydrogel lacks surface functional groups that can mediate endothelialization on the inner surface of a PVA vascular graft (Ino et al., 2013; Cutiongco et al., 2016a). In native blood vessels, an endothelial cell lining functions as a barrier between blood and smooth muscle cell layers, controlling the proliferation and migration of the smooth muscle cells by secreting enzymes and inhibitors to the enzyme (Allaire and Clowes, 1997). Endothelial cells also prevent the activation of platelets and subsequent thrombogenesis. Hence, having an endothelial cell lining on the inner graft surface is crucial to attain a high graft patency rate in the long term.

Previous *in vitro* cell adhesion studies with plasma treated PVA films have shown to improve endothelial cell attachment on the PVA treated with N_2_/H_2_, N_2_, O_2_, or Ar plasma (Ino et al., 2013; Cutiongco et al., 2016a; Jurney et al., 2018). These cell adhesion studies were conducted in a short term (2–6 days), and the plasma treatments were performed on flat surfaces or on the external surface of a tube. Plasma treatment of the internal surface of a small diameter PVA vascular graft has not yet been reported before. While plasma treatment has been demonstrated to be a promising modification to enhance surface properties of PVA, performing luminal plasma treatment could be technically challenging. To obtain homogeneous plasma modification in the tube lumen, the tube would require to be completely straight. To apply plasma treatment to a hydrogel material, the scaffold would need to be dehydrated. However, an unconstrained dehydration of a hydrogel graft would often introduce curvature or deformation, which would not lead to a completely straight tube as required, depending on the wall thickness distribution along the graft and constraints applied to the graft during dehydration. Moreover, the stability of endothelial cell adhesion on plasma treated PVA hydrogel vascular grafts has not yet been reported and needs to be considered prior to performing *in vivo* work.

In this study, we demonstrated that luminal plasma treatment of PVA hydrogel grafts internal surface can be performed. We hypothesized that using the luminal RFGD plasma treatment previously reported (Mantovani et al., 1999), we would functionalize the inner surface of small diameter PVA vascular grafts (4 mm inner diameter when hydrated, and ~3 mm inner diameter when dehydrated for plasma treatment). Moreover, it was hypothesized that the surface modification would be stable, and the surface would facilitate longer term maintenance of an endothelial cell layer without increasing platelet activation. Having stable grafted functional groups on the material surface is beneficial to achieve working off-the-shelf vascular grafts. To support clinical translatability, grafts were stored in an ambient storage condition until they were used for experiments.

## Materials and Methods

### PVA Graft Fabrication

PVA crosslinking was prepared as previously published (Chaouat et al., 2008). In brief, 10% (w/v) PVA (Aldrich, USA, Mw 85,000-124,000, hydrolysis percentage 87–89%) was mixed with 15% (w/v) sodium trimetaphosphate (STMP) (Aldrich, USA) for 5–10 min until a homogeneous solution was obtained. 30% (w/v) NaOH (Sigma-Aldrich, USA) was then added into a stirring solution and the solution was mixed for an additional 5–10 min. The solution was then centrifuged to remove any bubbles.

For an unpatterned PVA tube graft, a cylindrical rod coated with crosslinking PDMS (base: crosslinker = 10:1) (Dow Corning, USA, Sylgard 184), which was cured at 60°C. The PDMS coated rod was air plasma treated (85 W, 0.8 NL/h) for 80 s and was coated with 12 layers of crosslinking PVA solution (thickness 0.95 ± 0.17 mm). The PVA coated cylindrical rod was then kept in a cabinet at a controlled temperature of 20°C and controlled humidity of 70% for 3 days. Afterwards, the PVA coated cylindrical mold was rehydrated in 10 × phosphate buffered saline (PBS) (Fisher Scientific, USA) followed by 1 × PBS and deionized water. The PVA grafts were subsequently demolded from the cylindrical rod.

For a PVA graft with microtopography, a thin layer of micropatterned PDMS was made by spin coating 2 g of crosslinking PDMS (base: crosslinker = 5:1) on a patterned PDMS mold at 1,500 rpm for 15 s. The thin PDMS layer was rolled around the cylindrical rod and was air plasma treated as aforementioned. The rod was kept in crosslinking PVA solution and was sonicated for 90 min at 49 kHz. The cylindrical rod was then coated with layers of crosslinking PVA and it was kept in a cabinet as before. The rehydration of the PVA patterned tubes was done in 10 × PBS followed by 1 × PBS and deionized water. Patterned PVA grafts were subsequently demolded from the mold.

### PVA Film Fabrication

PVA crosslinking was prepared as previously described. The solution was then centrifuged to remove any bubbles and was poured into dishes. The dishes were kept in a cabinet with controlled temperature and humidity of 20°C and 70%, respectively, until the film was dried. The PVA film was then rehydrated in deionized water and demolded from the dishes.

To make a PVA film with microtopography, the crosslinking solution was poured on a dish with PDMS patterned mold. The dish was centrifuged at 600 rpm for 1.5 h and was then kept in a cabinet with controlled temperature and humidity as before until the PVA film was fully dried. The rehydration of patterned PVA film was done in 10 × PBS followed by 1 × PBS and deionized water.

### Scanning Electron Microscopy

PVA was dehydrated and coated with gold. Scanning electron microscope images were taken with a high-resolution field-emission scanning electron microscope (Zeiss 1550, Carl Zeiss AG, Oberkochen, Germany) at accelerated voltage of 7 keV.

### Luminal NH_3_ Plasma Treatment

**Figure 1:**
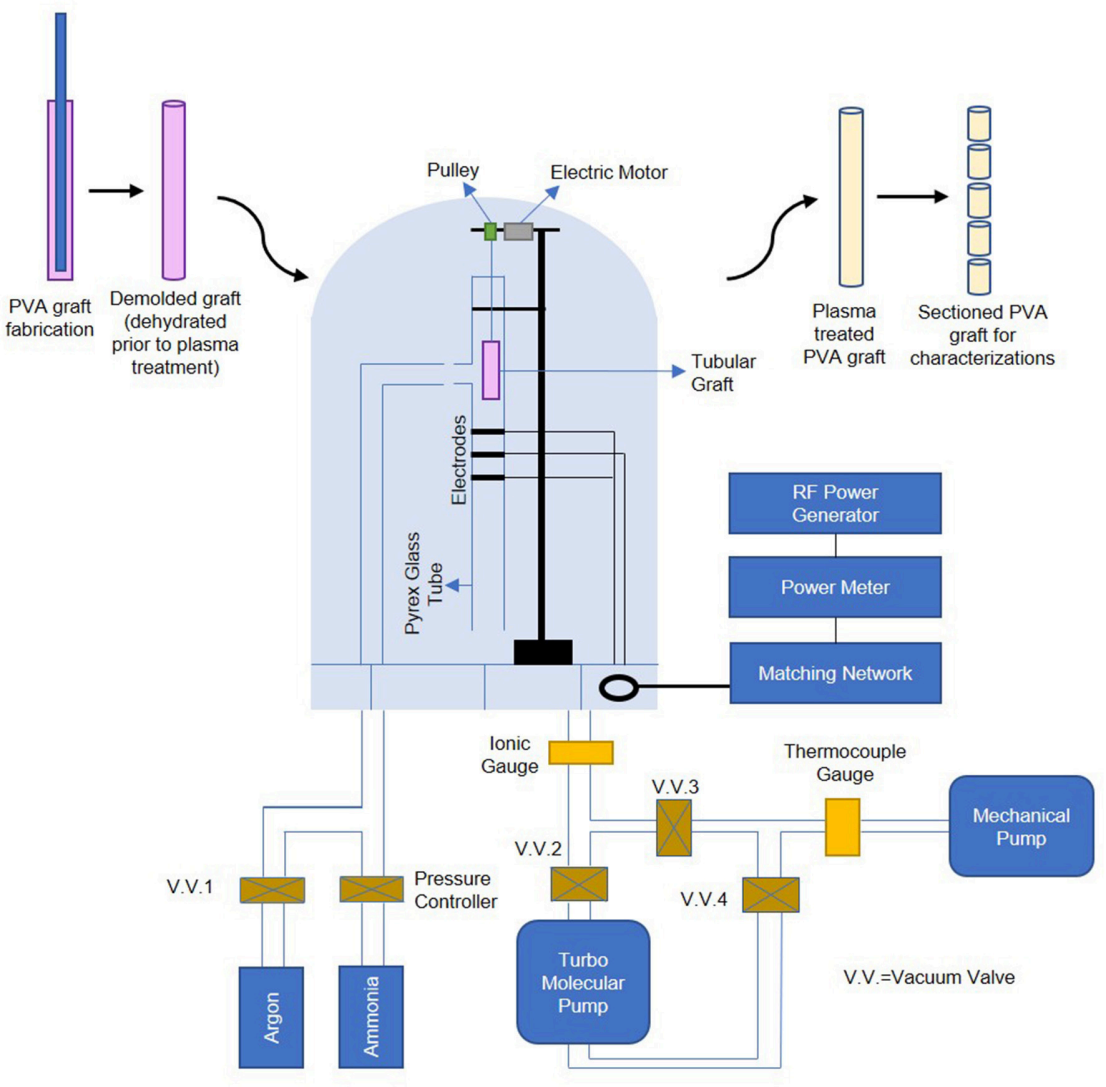
Schematic of PVA graft plasma treatment process, plasma treatment setup is adapted by permission from Springer Nature Customer Service Center GmbH: Springer, PLASMAS AND POLYMERS, Ammonia RF-Plasma Treatment of Tubular ePTFE Vascular Prostheses, Mantovani et al. (1999).

Luminal NH_3_ plasma treatment with the RFGD system was performed as previously described (Mantovani et al., 1999), and the setup is shown in Figure 1. Thin and straight cylindrical needles with diameter at least 1.25 mm smaller than the cylindrical molds used for PVA fabrication were inserted into hydrated PVA grafts' lumen. The whole assembly was then kept in a cabinet at a controlled temperature of 20°C and controlled humidity of 70% for slow dehydration; this step is crucial as it preserves the grafts' surface topography and maintains the graft ends to remain open and the graft body to remain completely straight during dehydration process. The dehydrated PVA graft (5.5–11 cm long, ~3 mm inner diameter) was then detached from the cylindrical needle and was inserted into a cylindrical Pyrex glass tube for luminal plasma treatment. Three capacitively coupled copper electrodes were located at the middle section of the tube for plasma generation. After pressure of < 5 × 10^−5^ Torr was reached, high purity NH_3_ gas was introduced into the chamber from the inlet which was 5 cm below the upper end of the tube. The NH_3_ plasma ignition was initiated by a radio-frequency generator (13.56 MHz) at a power of 65 W for 45 s, and thereby modified the luminal surface of the PVA graft. To plasma treat PVA films, the hydrated films were cut into 15.6 mm diameter circles (size of wells in a 24-well plate) and dried curve on a microcentrifuge tube wall. The dried PVA films were then assembled into a 4 mm diameter straw using small pieces of double-sided tape for plasma treatment. The plasma treated samples were stored in 15 ml polypropylene centrifuge tubes (VWR, China) and were kept on a lab bench until they were used for experiments.

### X-ray Photoelectron Spectroscopy (XPS) Measurement

The chemical composition of the surface was investigated by an XPS PHI 5600-ci spectrometer (Physical Electronics, Eden Prairie, MN). The main XPS chamber was maintained at a base pressure of < 8 × 10^−9^ Torr. An achromatic aluminum X-ray source (1486.6 eV) was used at 300 W with a neutralizer to record the survey spectra, and a standard magnesium X-ray source (1253.6 eV) was used to record high resolution spectra of C1s and day 77 N1s, without charge neutralization. The detection angle was set at 45° with respect to the normal of the surface and the analyzed area was 0.125 mm^2^. Measurements were done at equidistant positions along dehydrated PVA grafts. High resolution N1s spectra were generated with monochromated Al Kα 1486.6 eV, Thermo VG Scientific ESCALab 250 microprobe (WATLab, Waterloo) with charge compensation. The analytical chamber pressure was maintained at 2 × 10^−9^ mbar during measurement. The resulting high resolution N1s spectra were calibrated by 2.2 eV according to C1s spectra correction.

### Fourier-Transform Infrared Spectroscopy (FTIR)

FTIR was performed using Bruker Tensor 27 equipped with liquid nitrogen cooled mercury cadmium telluride (MCT) detector. Measurement was obtained with attenuated total reflection (ATR) mode with wavenumber ranges from 400 to 3,900 cm^−1^. Sixty-four scans were acquired at a spectral resolution of 4 cm^−1^. The depth of analysis was estimated to be 1 μm.

### Water Contact Angle Measurement

Unpatterned (UP) PVA and two other topographies (2 μm linewidth × 2 μm height × 2 μm spacing gratings (2 μG) and 1.8 μm diameter × 2 μm pitch concave lenses (CCL) tubes were used in the contact angle measurement. Plasma treated films were indicated with a letter P behind the abbreviation, i.e., UPP, 2 μGP, CCLP for plasma treated unpatterned, microgratings, and concave microlenses grafts, respectively. Water contact angle was measured on PVA films using an in-house built instrument. Films were hydrated during the test, and its surface was dabbed dry prior to test. The water volume used for measurement was 3 μl and images were captured within 10 s after the water drop was on the film surface. Three samples were measured for each experimental groups and each samples were measured once.

### Cell Adhesion Study

PVA grafts were cut longitudinally on one side and were exposed to UV for 20 min. Then, samples were incubated with 10% Penicillin/Streptomycin (Gibco, USA) and 1% Amphotericin-B (VWR, USA). PVA samples were then placed in 24-well plate wells with autoclaved silastic tubings (Dow Corning, USA, 0.375 inch inner diameter), yielding a seeding area of 0.7 cm^2^. Samples were rinsed thoroughly with 1 × PBS. Afterwards, PVA samples were incubated with fetal bovine serum (FBS) (Gibco, USA) and were centrifuged at 1,000 rpm for 30 min. PVA was kept in 2–8°C fridge overnight or in 37°C incubator for 30 min prior to cell seeding.

EA.hy926 (ATCC, USA) and primary HUVEC (Lonza, USA) cells used at passage number 7 were cultured according to ATCC and Lonza protocols, respectively. Confluent cells were passaged with 0.05% Trypsin/EDTA (Gibco, USA) and were seeded on the PVA at a seeding density of 50,000 cells/well. The endothelial cells were seeded onto films that were stored for at least 3–30 days before cell culture. The well plate was then spun down at 100 × g for 10 min to bring the cells down closer to the PVA substrate. Cell media change was performed daily, and cells were cultured for 2, 9, 12, 14, and 30 days for EA.hy926 and for 16 days (until cell monolayer was formed) for primary HUVEC before performing subsequent cell immunofluorescence staining.

### Cell Immunofluorescence Staining and Cell Number Quantification

Culture was washed with 1 × DPBS buffer with calcium (Gibco) prior to fixation with 4% paraformaldehyde (PFA) (Sigma Aldrich, USA) for 15 min. The fixed samples (both cell types) were then permeabilized by incubation with 0.05% Triton X-100 (Sigma, USA) and 50 nM glycine (Aldrich, USA) for 15–20 min. Cell nuclei and F-actin staining was done with DAPI (1:5,000) and Phalloidin (1:500) (Invitrogen, USA) incubation for 30 min, respectively. The samples were imaged using Zeiss immunofluorescence microscope at 10 × and 20 × magnification. Brightness and contrast adjustments were performed using ImageJ version 1.50i, Java 1.8.0 (developed by Wayne Rasband). Cell number quantification was performed through manual counting of the cell number from 4 to 12 images with 20 × magnification, or 3–9 images with 10 × magnification of different areas on the PVA surface. The cell number was then normalized to images area to give cell density values (cells/cm^2^).

### Stability Study of Plasma Treated PVA Surfaces

The assessment of treatment stability was done by performing XPS measurements (high and low resolution) on day 0, day 30, and day 77, water contact angle measurements on day 7, day 23, and day 60, and a cell adhesion study for a 30-day period. The stability studies with XPS and the cell adhesion study were performed on UPP PVA grafts (plasma treated), while the contact angle measurements for stability study were done on UPP PVA films (plasma treated). Cell adhesion studies were terminated for immunofluorescence staining with DAPI and phalloidin on day 2, 9, 12, 14, and 30 of culture.

### Platelet and Fibrin Accumulation Study With *ex vivo* Baboon Shunt Model

All baboons (*Papio anubis*) were housed and taken care of by Oregon National Primate Research Center (ONPRC) staff according to the “Guide to the Care and Use of Laboratory Animals.” Studies were approved by ONPRC Institutional Animal Care and Use Committee (IP00000300).

The *ex vivo* shunt study was achieved as previously described (Anderson et al., 2014). Prior to experiments, baboon platelets were labeled with 111-Indium and homologous fibrinogen was labeled with 125-Iodine. The baboon femoral artery and vein were accessed and extended with silicon tubing, to which the PVA graft was attached for 60 min without anticoagulant or anti-platelet administration. The platelet accumulation was measured by gamma camera at increments of 5 min. The graft was then flushed, and fibrinogen quantification occurred after a complete decay of the 111-Indium. Data were obtained from four different animals.

### Statistical Analysis

Data are presented as mean ± standard error, except for *ex vivo* shunt data, where data are presented as mean ± standard deviation. Throughout significance is defined as *p* < 0.05. In figures, ^*^ denotes statistical significance with *p*-value of ≤ 0.05, ^**^p-value ≤ 0.01, ^***^p-value ≤ 0.001, and ^****^p-value ≤ 0.0001. Number of replicates is reported in individual figure legends.

Statistical comparisons between experimental groups for surface nitrogen content, water contact angle, surface composition, and cell adhesion were completed using GraphPad Prism 6. Differences in XPS element percentages and high resolution C1s peak percentages were computed using one-way ANOVAs and Sidak analyses with 95% confidence interval, whereas differences in O/C and N/C were computed with two-sided and one-sided unpaired *t*-test, respectively. Differences in %N along graft surfaces to analyze surface homogeneity were computed with two-way ANOVA and Tukey's *post-hoc* analysis with 95% confidence interval. Further, differences in water contact angle and HUVEC cell number quantification were computed with one-way ANOVA and Tukey's *post-hoc* analysis with 95% confidence interval. Additionally, differences in EA.hy926 cell number on day 12 were computed with unpaired *t*-test (two-sided) and 95% confidence interval.

Statistical comparisons of platelet and fibrin accumulation on PVA samples in *ex vivo* shunts were calculated using R (R foundation for statistical computing, version 3.5.2) packages nlme (version 3.1–137) and multcomp (version 1.4-8). While ePTFE and collagen-coated ePTFE controls are displayed as a reference in figures, only PVA samples (*N* = 6–7 for each surface modification) were included in statistical models. Platelet and fibrin data were natural-log transformed prior to analysis in order to more closely approximate normal distributions. Transformed real-time platelet adhesion data were analyzed with a multi-way repeated measures ANOVA with factors of time, patterning, and plasma treatment over the entire 60 min time course. Transformed endpoint fibrin data were analyzed with a two-way ANOVA against patterning and plasma treatment. Patterning levels were unpatterned, gratings, and concave. Plasma levels were no plasma, fresh plasma treatment (tested 7–11 days post-treatment), and aged plasma treatment (tested 44–60 days post-treatment). Statistically significant main effects were analyzed with a Tukey's *post-hoc* as appropriate.

## Results

### Surface Characterization of Plasma-Treated PVA Luminal Surface

**Figure 2:**
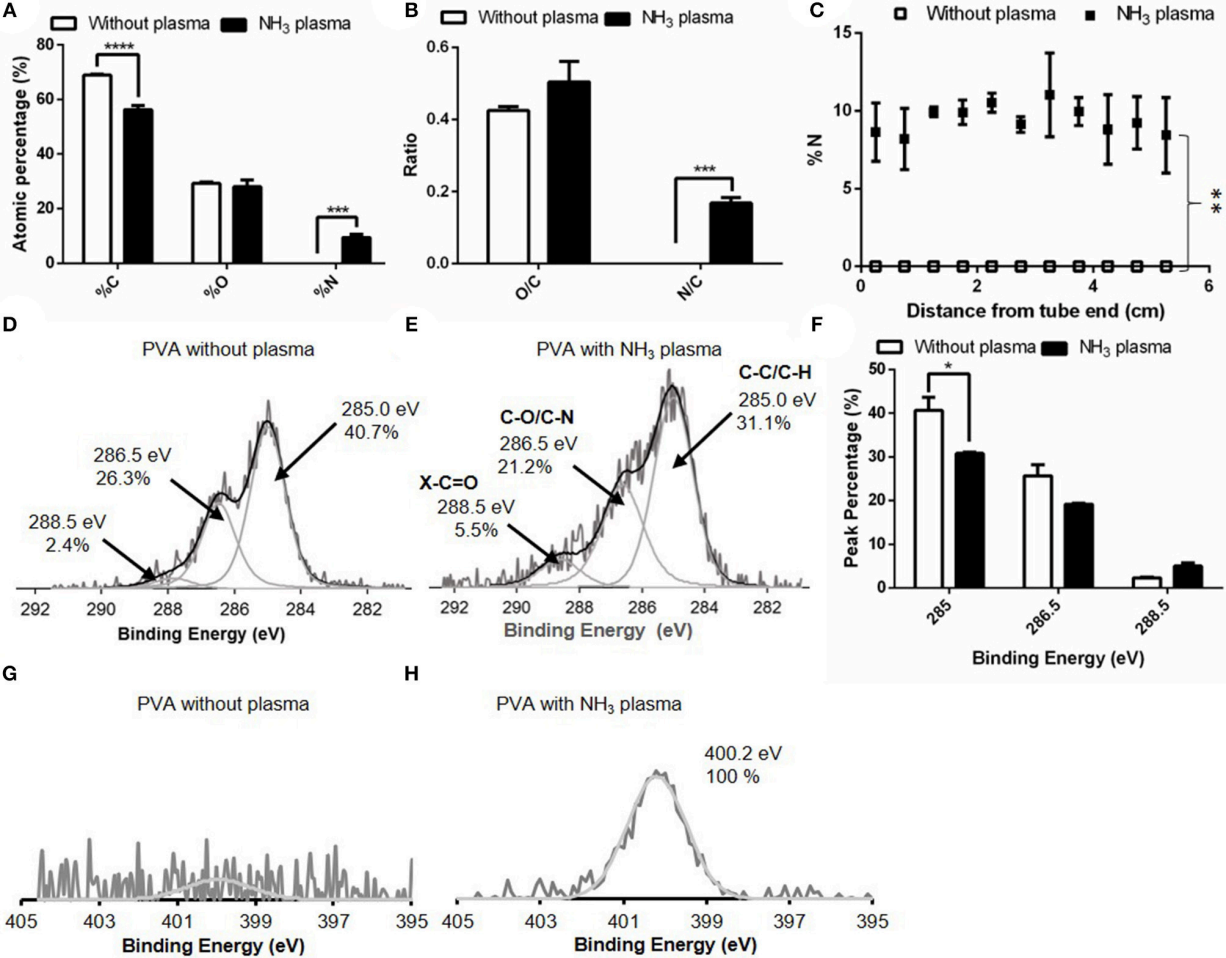
Comparison of surface elements without and with NH_3_ plasma as measured with X-ray photoelectron spectroscopy (XPS). **(A)** XPS survey for %C, %O, and %N of PVA luminal surface and **(B)** O/C and N/C ratio calculated from **(A)**. **(C)** %N as measured at 11 equidistant positions along the 5.5 cm-long grafts. *N* = 3 grafts for experimental groups in **(A–C)**. High resolution C1s spectra on PVA grafts **(D)** without plasma and **(E)** with NH_3_ plasma. **(F)** Comparison of peak percentage of the C1s spectra at binding energy 285, 286.5, and 288.5 eV before and after plasma treatment. *N* = 2 grafts for both experimental groups. High resolution N1s spectra on PVA grafts **(G)** without plasma and **(H)** with NH_3_ plasma. ^*^ denotes statistical significance with *p* ≤ 0.05, ^**^p ≤ 0.01, ^***^p ≤ 0.001, and ^****^p ≤ 0.0001.

The atomic percentages of C, O, and N on untreated and NH_3_ plasma treated PVA grafts were shown in Figure 2A. The plasma surface modification inducted by NH_3_ of the luminal surface of PVA successfully resulted in a significant increase in the nitrogen percentage (*p* ≤ 0.001, *N* = 3 grafts for both untreated and plasma treated groups), as measured with XPS. The atomic percentages of C, O, and N on untreated PVA surfaces were 68.9 ± 1.8, 29.2 ± 1.9, and 0.0 ± 0.0%, respectively, while the atomic percentages of C, O, and N after plasma treatment were 56.3 ± 3.5, 27.9 ± 4.1, and 9.5 ± 2.5%, respectively. The significant increase in nitrogen was also shown in the N/C ratio which was calculated from the survey XPS, while there remained no significant change in O/C after plasma treatment (Figure 2B). The homogeneity of surface modification was further characterized by measuring the nitrogen percentages of the plasma treated PVA surface at 11 equidistant positions along the 5.5 cm long graft. There were no statistical differences found between the nitrogen percentages along the treated grafts (*N* = 3) (Figure 2C). The nitrogen percentage values averaged at 9.4%, while on the PVA luminal surface without plasma treatment, there was no nitrogen element detected (*N* = 3). This increase in nitrogen percentage was statistically significant with *p* ≤ 0.01 compares to the untreated control. A longer (11 cm) PVA graft was also successfully plasma treated with this technique and the nitrogen atomic percentage from the graft end to 6.4 cm away from the end was shown in Supplementary Figure 1. In order to identify the surface functional group before and after the treatment more specifically, high-resolution XPS measurements of C1s and N1s were performed. Compared to the untreated PVA surface, the plasma treated PVA demonstrated an increase in carbonyl functional group (C = O) percentage, whereas the C-O/C-N functional group percentage was lower (Figures 2D,E). The overall C-C/ C-H bond percentage was also reduced due to the increase in other bond percentages. The peak percentages of the high resolution C1s spectra were calculated before and after the plasma treatment and this decrease in C-C/C-H peak was found to be statistically significant (*p* ≤ 0.05, *N* = 2 grafts for both groups) as shown in Figure 2F. The high-resolution spectra of N1s (measured on day 16 post-plasma treatment) showed the presence of nitrogen species at 400.2 eV on the graft inner surface compared to the untreated control (Figures 2G,H).

**Figure 3:**
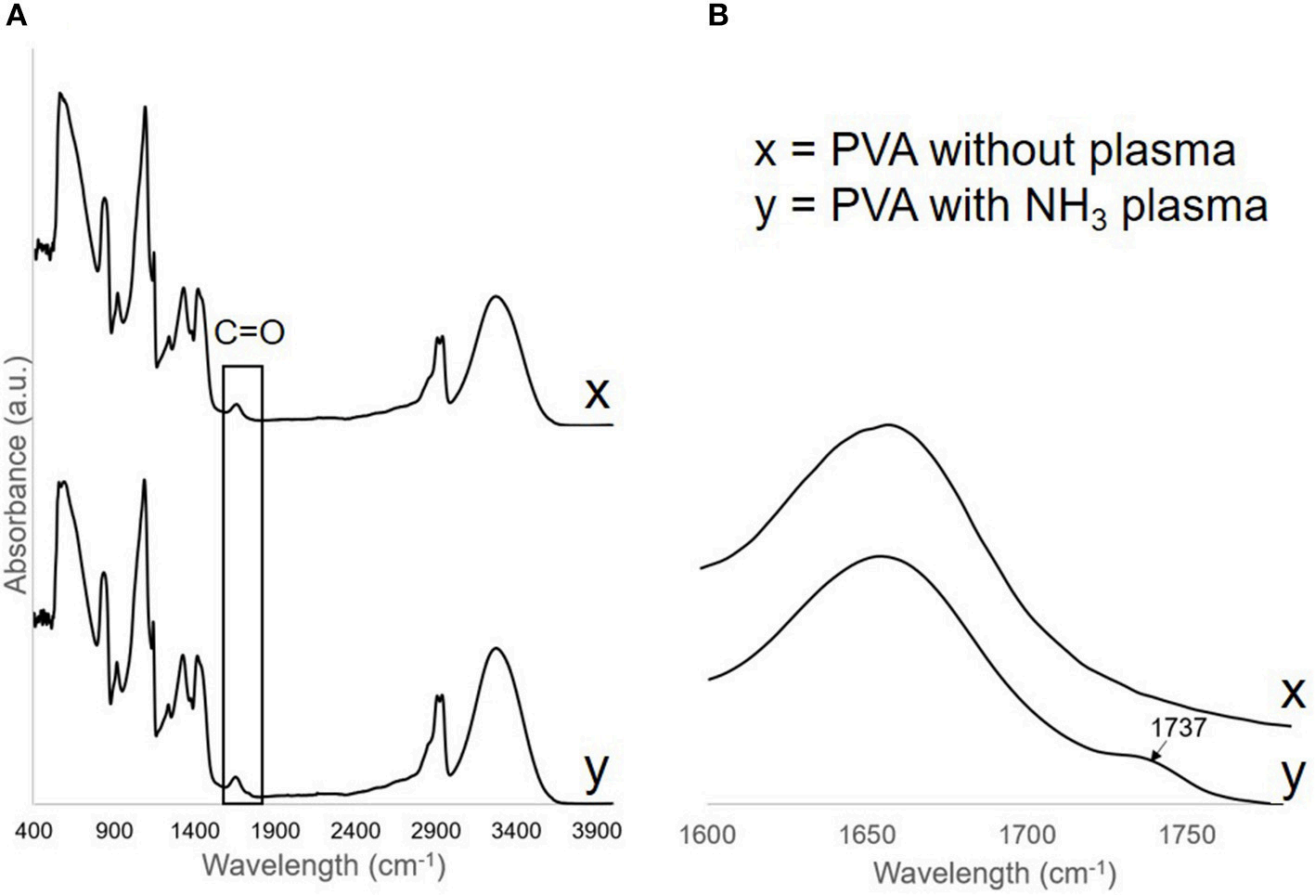
**(A)** FTIR spectra of PVA without plasma, x, and PVA with NH_3_ plasma, y. C = O peak was found on the PVA surface after plasma treatment and was shown more clearly in **(B)**.

To confirm the change in the surface functional group after the treatment, FTIR spectra were obtained from both treated (day 24 post-plasma treatment) and untreated PVA grafts. On the plasma treated PVA graft surface, we observed a C = O peak at wavelength 1,737 cm^−1^ which was not observed on the untreated PVA spectra (Figure 3). All other peaks remain the same before and after the treatment.

### Changes in PVA Surface Wettability Before and After Plasma Treatment

**Figure 4:**
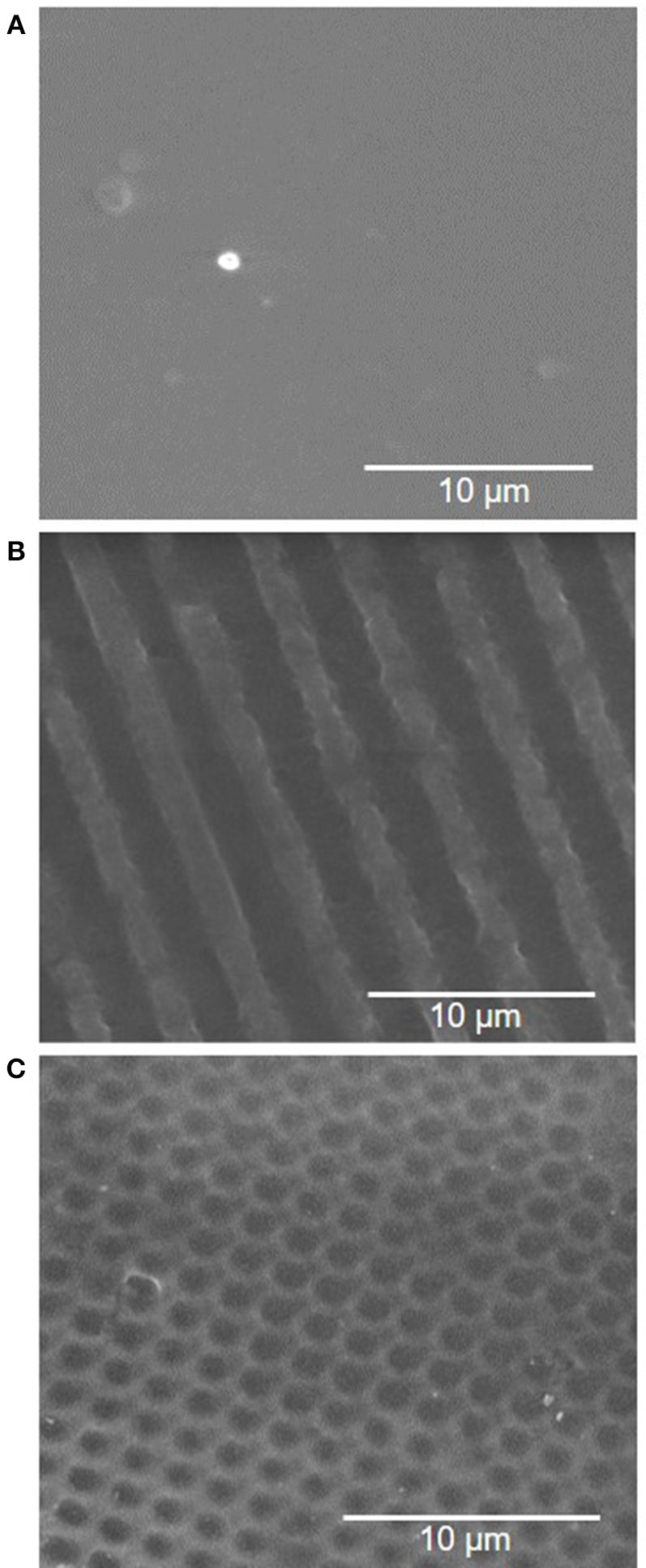
Scanning electron microscopy (SEM) images of PVA luminal surface with **(A)** no pattern, **(B)** 2 μm gratings, and **(C)** 1.8 μm concave lenses topography.

**Figure 5:**
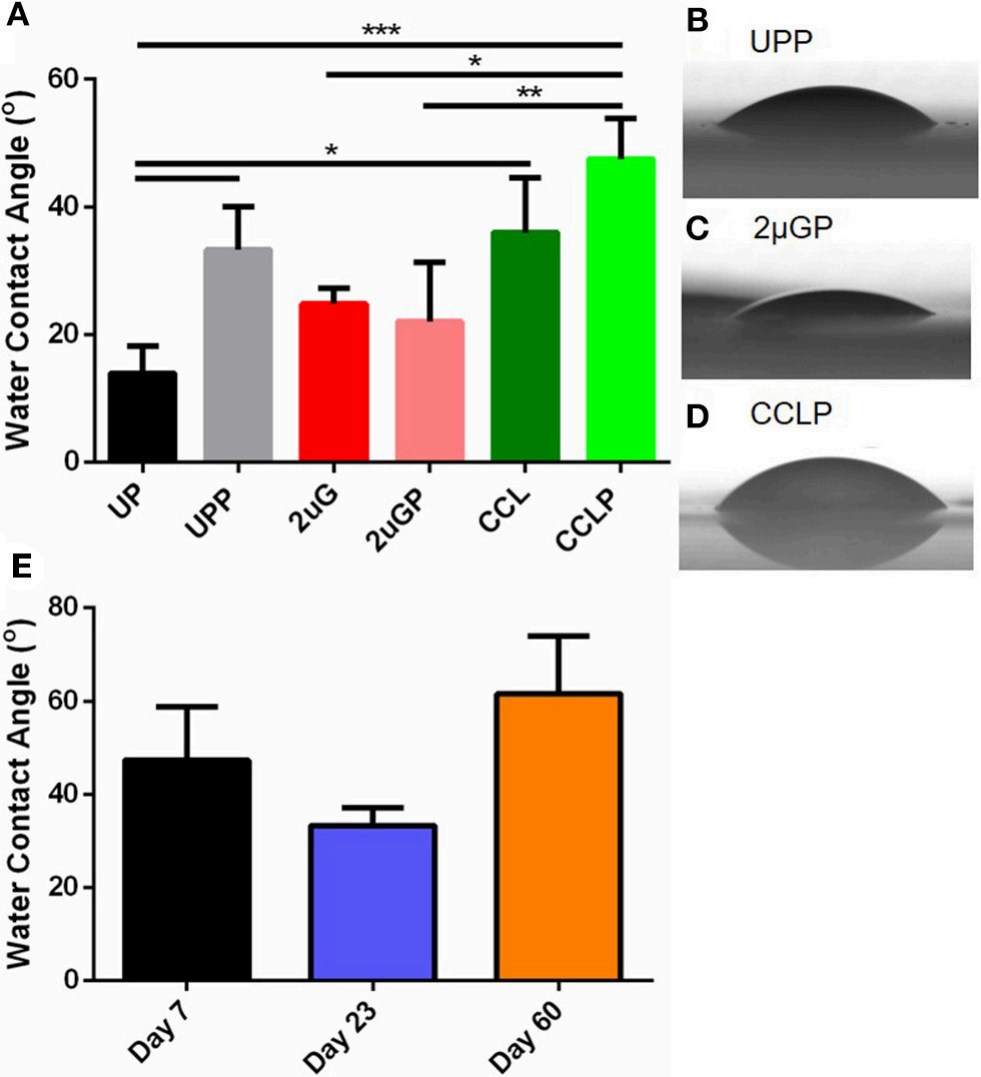
**(A)** Comparison of water contact angle measurement before and after NH_3_ plasma treatment on unpatterned (UP), 2 μm gratings (2 μG), and 1.8 μm concave microlenses (CCL) PVA films. Suffix *P* indicates plasma treated samples (e.g., UPP, unpatterned and plasma treated PVA). Representative images of the water droplets on plasma treated PVA surfaces were shown for **(B)** unpatterned **(C)** 2 μm gratings, and **(D)** 1.8 μm concave microlenses samples. **(E)** Water contact angle of plasma treated unpatterned PVA measured on day 7, day 23, and day 60 post-plasma treatment. *N* = 3 for all experimental groups in **(A,E)**. ^*^Denotes statistical significance with *p* ≤ 0.05, ^**^p ≤ 0.01, ^***^p ≤ 0.001.

Surface wettability can affect protein adsorption and subsequent cell attachment on a biomaterial surface; therefore, the water contact angles of the plasma treated PVA films were measured. As previous study showed the effectiveness of microgratings and concave microlenses topography in promoting improved cell attachment and alignment on the PVA surface (Cutiongco et al., 2016a), three topographies were studied: unpatterned (UP) surface (Figure 4A), 2 μm gratings (2 μG) topography (Figure 4B), and 1.8 μm concave lenses (CCL) topography (Figure 4C). The surfaces were characterized with SEM and were found to be in good fidelity. It was shown that the surface hydrophilicity on the plasma treated PVA surfaces was reduced. The decrease in hydrophilicity was observed on both UPP (*p* ≤ 0.05 against UP control, *N* = 3) and CCLP surfaces (no significance against CCL control, *N* = 3) (Figure 5A). The average water contact angle of the 2 μGP topography was, however, slightly decreased after the plasma treatment and possessed a larger standard deviation compared to the 2 μG PVA. The images of water droplets on plasma treated surfaces are shown in Figures 5B–D.

The stability of the plasma treated surface after storage in ambient conditions was first investigated with water contact angle measurement of the UPP PVA. Water contact angle measurements were taken on day 7, 23 and 60 on plasma treated PVA films. The water contact angle was found to be lower on day 23 (33.3 ± 6.8°, *N* = 3 films) and higher on day 60 (61.6 ± 21.5°, *N* = 3 films) compared to day 7 measurements (47.4 ± 19.8°, *N* = 3 films). However, these differences were not found to be statistically significant (Figure 5E).

### Characterization of Grafted Functional Groups up to 77 Days After Plasma Treatment

**Figure 6:**
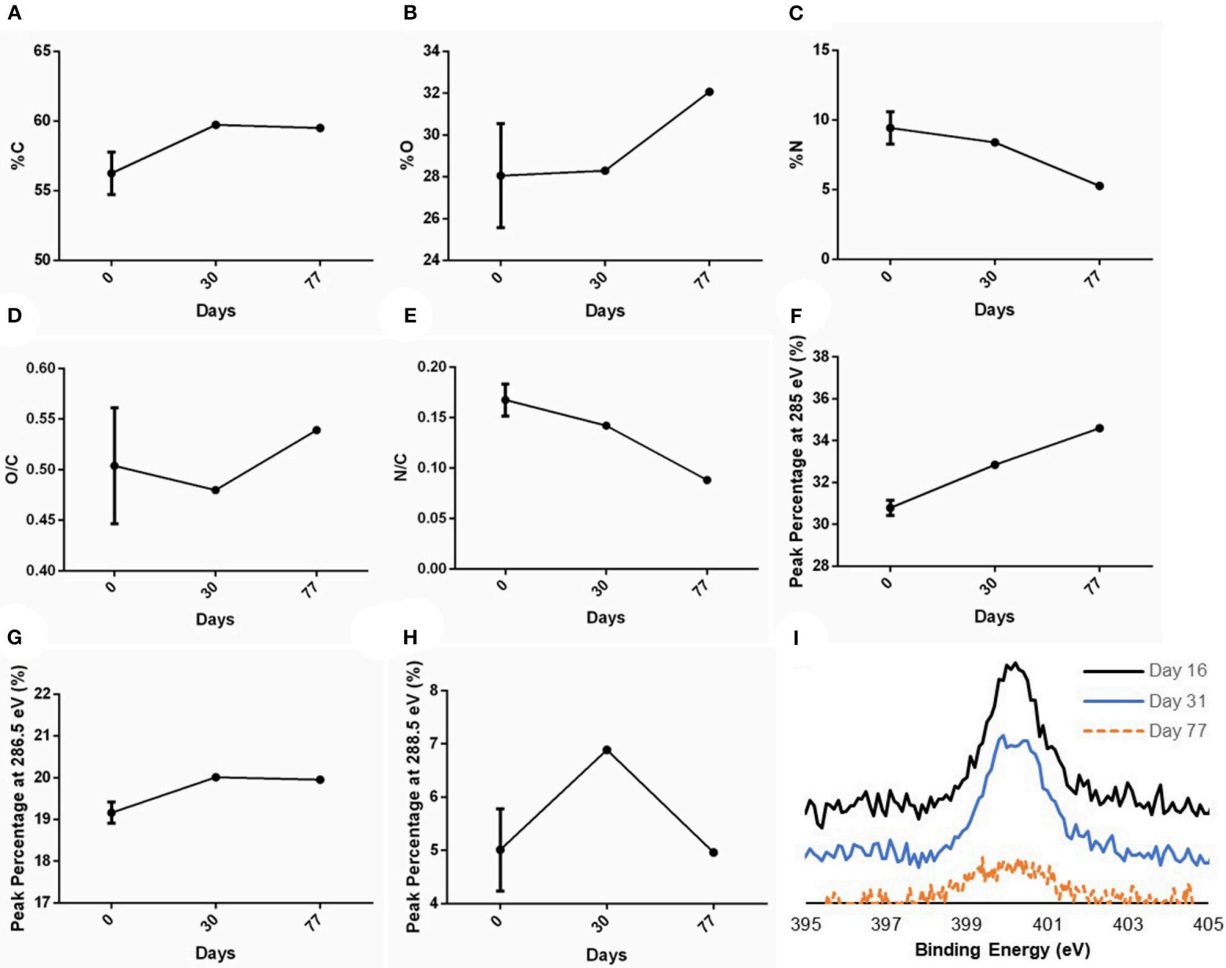
XPS measurements of C, O, and N after graft storage in ambient conditions. XPS survey of PVA grafts performed on day 0 (right after plasma treatment), day 30 and 77 post-plasma treatment for **(A)** %C, **(B)** %O, **(C)** %N, and the calculated ratio of **(D)** O/C and **(E)** N/C based on the XPS survey. The peak percentage of the peaks at binding energy **(F)** 285 eV, **(G)** 286.5 eV, and **(H)** 288.5 eV of the high resolution C1s spectra were also measured on day 0, 30, and 77. In **(A–E)**, *N* = 3 grafts, 1 graft, and 1 graft for day 0, 30, and 77 measurements, respectively. In **(F–H)**, *N* = 2 grafts, 1 graft, and 1 graft for day 0, 30, and 77 measurements, respectively. Multiple points were measured in each sample; refer to Supplementary Table 1 for the data for each sample. **(I)** High resolution N1s spectra measured on day 16, 31, and 77 post-plasma treatment.

The stability study of the surface modification of samples stored in ambient conditions (25°C temperature and 60% average humidity) was examined on both plasma treated and untreated PVA surfaces. Day 0 denotes that the measurement was taken right after plasma treatment was performed. XPS measurements were taken right after graft plasma treatment, at 1- and 2-month time points after plasma treatment. A slight drop in both %N and N/C was observed in the day 77 measurement which caused a slight increase in both %O and O/C, while %C remained at 59.7% on day 30 and 59.5% on day 77 (Figures 6A–E). Using the curve-fit peak areas, the peak percentages of peaks at 285, 286.5, and 288.5 eV of the total C1s spectra were also measured at the three time points. The peak percentages at binding energy 285, 286.5, and 288.5 eV on day 0 were 30.79 ± 4.00%, 19.16 ± 4.40%, and 5.01 ± 1.35% (*N* = 2 grafts), respectively, while the peak percentages on day 30 were 32.85, 20.01, and 6.89% (*N* = 1 graft) as shown in Figures 6F,G. Additionally, the peak percentages on day 77 were 34.60, 19.95, and 4.96% (*N* = 1 graft) as shown in Figure 6H. While the peak percentage at 285 eV showed a slight increase on both day 30 and 77 measurements, the peak percentages at both 286.5 and 288.5 eV remained close to the day 0 measurements. Further, the high resolution N1s spectra showed the presence of nitrogen species on day 16, 31, and 77 (Figure 6I).

### *In vitro* Endothelial Cells Attachment and Cell Layer Maintenance Up to 30 Days of Culture

**Figure 7:**
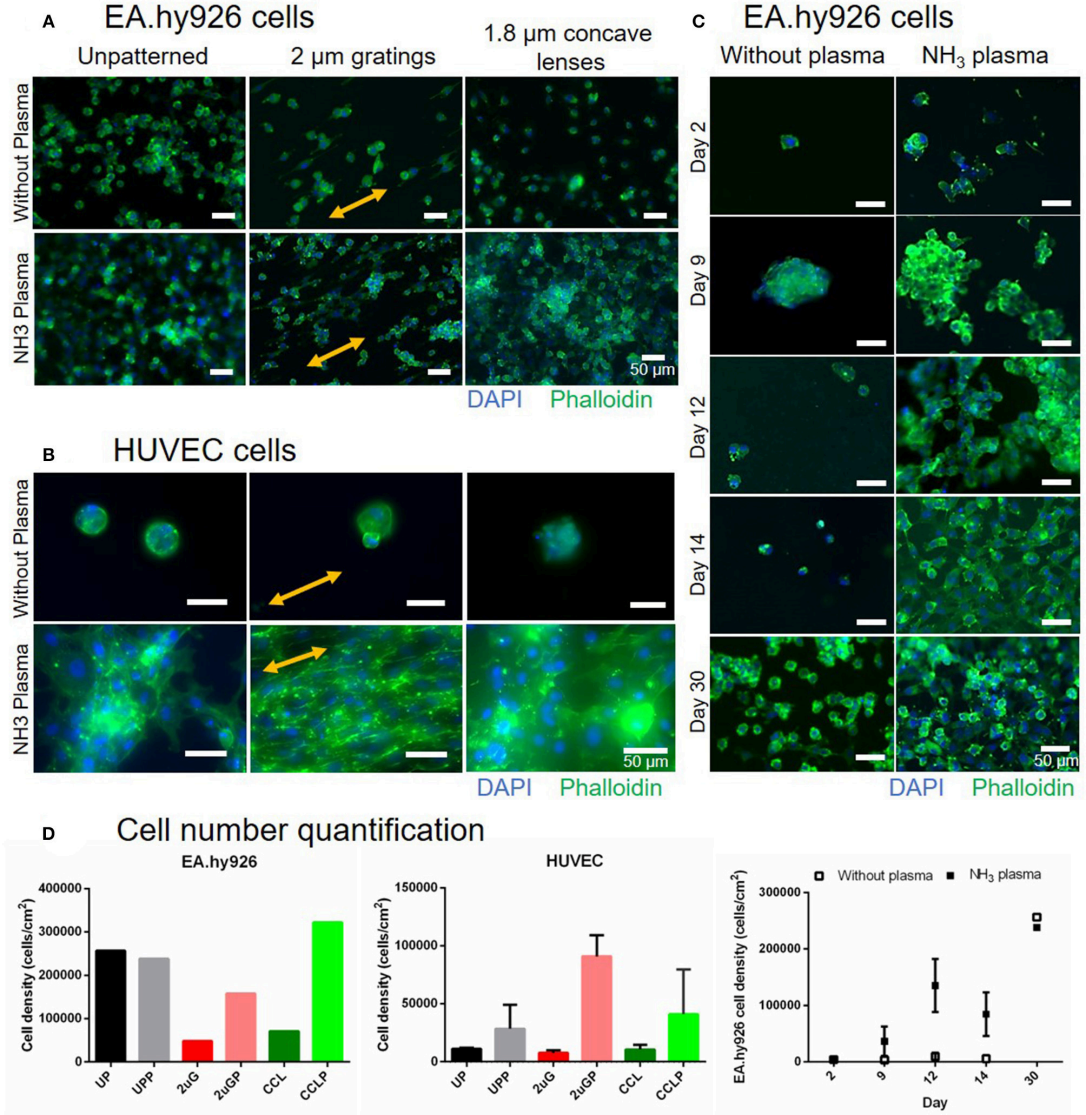
Comparison of EA.hy926 and primary human umbilical vein endothelial cell (HUVEC) cell attachment on PVA surfaces without plasma treatment and with NH_3_ plasma treatment. **(A)** Fluorescence images of EA.hy926 cells fixed on day 30 of culture and of **(B)** HUVECs fixed on day 16 of culture on unpatterned and patterned PVA. Nuclei and F-actin were stained blue and green, respectively. Double-headed arrows indicate gratings direction. **(C)** The stability of cell adhesion on the PVA surfaces with and without plasma treatment was further studied and compared on unpatterned sample up to 30 days of culture. Cells were almost confluent on day 14 on plasma treated surfaces and maintained their attachment as observed on day 30. Scale bar: 50 μm. **(D)** Cell number quantification of EA.hy926 cells (day 30, *N* = 1 for each groups), HUVECs (day 16, *N* = 2 for each groups), and stability study with EA. hy926 cells (day 2, 9, and 14, *N* = 1 and 2 for without plasma and NH_3_ plasma groups, respectively; day 12, *N* = 2 and 3 for without plasma and NH_3_ plasma groups, respectively; and day 30, *N* = 1 for each groups).

To assess the effectiveness of luminal NH_3_ plasma treated PVA grafts in facilitating *in vitro* cell adhesion, cell adhesion studies were performed using endothelial EA.hy926 and primary HUVEC cells on 7- and 3-day old plasma treated samples, respectively, both UP and patterned surfaces with 2 μG and CCL topographies. The EA.hy926 and HUVEC cultures were maintained for 30 and 16 days, respectively, before fixing and staining (Figures 7A,B). Improved EA.hy926 cell attachment was observed on the plasma treated surfaces, mainly on patterned surfaces (Figures 7A,D, and Supplementary Table 2). The EA.hy926 did not show much difference in cell morphology on samples with and without plasma treatment and cells were more elongated on the 2 μG and 2 μGP topography. In contrast, HUVEC attachment improved in all the plasma treated samples, especially on the 2 μGP sample where endothelial monolayer was formed. On the plasma treated samples, HUVECs have a better cell spreading compared to the untreated controls where cells were observed to be in clumps. HUVEC cell alignment was clearly observed on the 2 μGP sample. Furthermore, EA.hy926 cells were fixed and imaged on day 2, 9, 12, 14, and 30 of culture (Figure 7C). The endothelial culture on the plasma treated surfaces showed improved cell attachment compared to the untreated control on day 2, 9, 12, and 14. On day 14 of culture, endothelial cells reached 90% confluency and the cell attachment was maintained up to day 30. The corresponding cell number quantification can be found in Figure 7D and Supplementary Table 2. However, there was no statistical difference between the experimental groups.

### Platelet and Fibrin Accumulation as Studied With *ex vivo* Baboon Shunt Models

**Figure 8:**
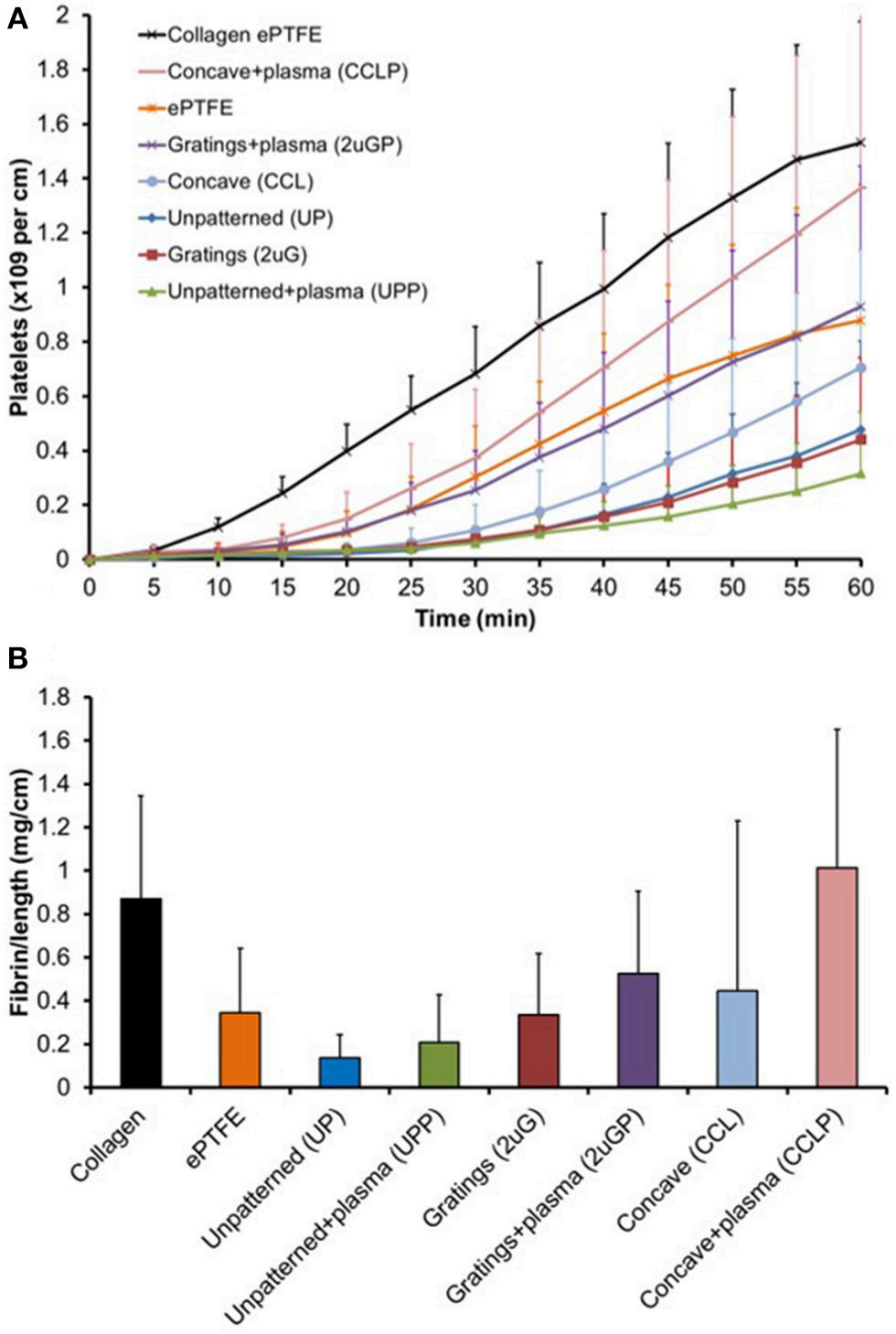
Untransformed shunt data for biological interpretation. **(A)** Platelet accumulation on PVA graft surface after exposure to whole, flowing blood without anticoagulants in the *ex vivo* shunt model. Statistical analysis performed with repeated measures multifactor ANOVA on 5-min time point data. **(B)** Normalized fibrin accumulation amount after 60 min on PVA graft surface. Statistical analysis was performed with multi-way ANOVA with factors of time, patterning, and plasma treatment (*N* = 6–7 samples for PVA experimental groups).

The platelet accumulation on both plasma treated and untreated PVA grafts was measured every 5 min over 1-h duration of study (Figure 8A). Eight different types of vascular grafts were tested: UP, UPP, 2 μG, 2 μGP, CCL, and CCLP PVA grafts, ePTFE grafts (untreated), and collagen coated ePTFE grafts (untreated). The highest platelet accumulation was observed on the collagen coated ePTFE which served as the positive control, whereas UP and UPP, 2 μG, and CCL PVA grafts demonstrated low platelet activation.

Untransformed shunt data are shown in Figure 8 for better biological interpretation, but statistics were performed on log-transformed data as described in the methods section. A multi-way repeated measures ANOVA found significant effects of both plasma treatment (*p* = 0.004) and material patterning (*p* = 0.011) on platelet attachment. Concave microlens patterning significantly increased platelet accumulation from unpatterned samples (*p* = 0.004), while microgratings patterning did not significantly alter platelet attachment. Fresh plasma treatments (day 7–11 samples) showed a significant increase in platelet attachment compared to no plasma treatment (*p* = 0.006); however, this was not reflected when the plasma treatment was aged (day 44–60 samples, *p* = 0.195). The change in platelet accumulation between fresh and aged plasma is shown in Supplementary Figure 2.

Plasma modification did not significantly alter fibrin accumulation in the two-way ANOVA (*p* = 0.386), but patterning was a significant factor (*p* = 0.033). In the *post-hoc*, concave patterning showed a significant increase in fibrin accumulation (Figure 8B, *p* = 0.034) compared to unpatterned samples similar to the platelet data; however, microgratings did not differ significantly from unpatterned samples (*p* = 0.22).

## Discussion

The challenge of plasma treating a small diameter tube structure is in achieving a sufficient amount of plasma to go through the small lumen in order to interact with the inner tube surface since plasma particles quench as they collide with the tube wall (Cao et al., 2007). A slight constriction or deformation of the tubular wall will result in plasma not being able to enter and modify the tubular structure. In this regard, care was taken when dehydrating PVA grafts prior to plasma treatment to ensure that the grafts were completely straight. A few studies have revealed that luminal plasma treatment can be accomplished (Hatada et al., 1982; Matsuzawa and Yasuda, 1984; Lin and Cooper, 1995; Kaibara et al., 2000; Cao et al., 2007; Qiu et al., 2017), and at least two studies performed the treatment for a small diameter polyethylene tubes (1–3 mm inner diameter and 46.5–60 cm long) by utilizing electrode movement outside the plasma reactor (Lin and Cooper, 1995; Cao et al., 2007). Nevertheless, none of these studies used hydrogel-based material, while hydrogel is known to have suitable mechanical properties for tissue engineering applications (Drury and Mooney, 2003; Yates et al., 2007; Abidian et al., 2012; Arslantunali et al., 2014; Cutiongco et al., 2016b). PVA hydrogel lacks surface functional groups to mediate endothelialization, while the patency rate of the currently available small diameter vascular graft (inner diameter < 6 mm) is low due to early thrombogenesis in the absence of an endothelial cell lining on the graft surface (Tordoir et al., 1995; Kirkwood et al., 2011). Therefore, this study compared the effects of the surface functionalization through luminal plasma treatment of small diameter PVA grafts to untreated PVA grafts and assessed the stability of the plasma treated surface.

The significant increase in atomic percentage of N as detected by the survey XPS was an indication of successful luminal plasma treatment of 3 mm dehydrated PVA grafts. In addition, the surface modification was homogeneous along 5.5 cm long grafts as there were no significant differences found between the measured *N* percentage values along the grafts, which is notable given that the grafts were made of hydrogel material. XPS results showed a significant increase in N/C (*p* ≤ 0.0001) between untreated and NH_3_ plasma treated samples. Further, an increase in carbonyl groups was also detected with the high resolution XPS of C1s, which could be coming from amide, ester, or carboxylic acid groups on the PVA surface. The increased ratio between C-O/C-N peak and C-C/C-H peak after the treatment could mean that amine functional groups could also be grafted on the PVA surface. However, as the nitrogen species peak binding energies are close to one another, these peaks overlap, and the differentiation of the nitrogen species cannot be easily done with XPS.

Since the grafts were stored in ambient conditions with air humidity of 50–70%, water vapor in the surrounding air can easily react with the nitrogen species such as amine to produce alkyl ammonium ion group and changing the surface composition of the treated PVA grafts. Nevertheless, on day 77, the nitrogen species was still present on the grafts as detected with XPS. Moreover, the peak percentages at 286.5 and 288.5 eV on day 30 and 77 measurements remained at similar level to day 0 measurement which indicated that the change if surface functional groups during storage, if any, was not remarkable.

While XPS could measure up to ~5 nm depth, FTIR could analyze up to 1 μm depth. Consistent to the XPS measurement, a weak carbonyl (C = O) peak was found on the FTIR absorbance spectra measured on day 24 post-plasma treatment of PVA grafts, but not on the untreated grafts. However, since amide peak was not observed on the spectra, the carbonyl peak on the spectra could be mainly detected from ester or carboxylic acid groups which may be more dominant throughout the 1 μm depth of FTIR analysis. Also, the weak peak could mean that the functional groups were present mainly at the graft surface. At the depth of FTIR analysis, the plasma treatment did not seem to change the main chemical structure of the PVA hydrogel as all other absorbance peaks were present on the plasma treated PVA grafts spectra as they were on the untreated PVA grafts spectra.

Compared to microwave (MW) plasma, radio frequency (RF) plasma has an advantage of having a more controlled ion bombardment on the surface (Klemberg-Sapieha et al., 1991), and is therefore a more suitable technique for plasma treating a tube structure despite possessing a lower electron density compared to MW plasma (Moisan and Wertheimer, 1993). In this study, we observed that the N/C ratio immediately after RF NH_3_ plasma treatment was significantly higher (*p* ≤ 0.05) compared to the microwave N_2_/H_2_ plasma treatment on the flat PVA surfaces previously reported (Ino et al., 2013). Thus, the RF plasma treatment reported herein has adequately grafted nitrogen functional groups on the internal surface of PVA grafts.

The water contact angle was found to be higher on both UPP and CCLP PVA, which was likely caused by the presence of a less polar, nitrogen containing functional group on the plasma treated surfaces compared to the polar hydroxyl functional group. This observation agrees with Cutiongco et al. (RIE N_2_ plasma on PVA) (Cutiongco et al., 2016a), but not so with Ino et al. (microwave N_2_/H_2_ plasma on PVA) (Ino et al., 2013), or Tusek et al. (microwave NH_3_ plasma on polyamide 6 foils) (Tušek et al., 2001). As water contact angle is mostly affected by surface roughness and functional groups, the discrepancy could be sourced from the difference in roughness of untreated PVA surfaces as well as the surface functional groups prior to the plasma treatment. In this study, the surface roughness was kept consistent by testing the contact angle of the surface that faced the PDMS mold for both UP and patterned PVA samples. We previously observed that the surfaces of PVA films that faced PDMS mold were much smoother than those that faced the air during the crosslinking process (data not shown). The average contact angle of 2 μGP PVA was slightly decreased with a larger variation in the measurements. We speculate that the plasma modification could have affected the topography sharpness which could result in a larger contact angle variation.

Cell-material interaction is facilitated by cell membrane receptors called integrins which are collectively known as focal adhesion (Kanchanawong et al., 2010). Improvement in cell adhesion on a material surface can therefore be achieved by providing ligands to the integrin which include amino and carbonyl groups on the biomaterial surface. Ammonia plasma was chosen to fulfill this role. The grafted nitrogen species on the plasma treated PVA grafts' surface, as detected by XPS and FTIR, contributed to the improved endothelial cell adhesion. In addition, because the plasma treatment caused an increase in water contact angle of the plasma treated PVA (PVA became less hydrophilic), this could create hydrophobic-hydrophobic interactions between material surface and proteins which were present in the serum during the sample serum incubation as well as in the cell media. All these factors act synergistically to result in a stable endothelial-cell lining on the plasma treated PVA surface for up to 30 days of culture. Likewise, both EA.hy926 and primary HUVEC cell attachment on patterned PVA surfaces was improved after the plasma treatment. The HUVECs on the plasma-treated PVA showed better cell-spreading compared to the untreated controls and were able to achieve monolayer formation on the 2 μGP samples. In contrast, the endothelial monolayer was unable to be formed in the case of EA.hy926 cells even after 30 days of culture. This observation agreed with a study reported before where the expression of adhesion molecule were significantly lower for the EA.hy926 cells as compared to primary HUVEC cells (Lidington et al., 1999). It was noted that the cell numbers on the day 30 untreated controls were very high compared to the untreated controls at other timepoints. Although the cause of this anomaly was still uncertain, we speculate that the highly proliferative nature of EA.hy926 cell line could contribute to the cell line survival in the absence of sufficient extracellular matrix proteins. Nevertheless, the addition of the nitrogen functional groups in the plasma treated PVA appeared to provide prolonged and more stable support of the cell adhesion on the hydrogel up to the 30-day culture period as examined in this study.

The presence of an endothelial cell lining in the graft lumen could prevent thrombosis and improve vascular graft patency. However, in *in vivo* environments, platelets' integrins may attach to the added surface functional groups and activate the platelets before an endothelial cell layer is established. Once a platelet is activated in this fashion, platelet accumulation may occur, which ultimately leads to thrombosis. In addition to platelets, fibrin is involved in the blood clot formation by forming a mesh-like structure and strengthening the clot structure. A hemocompatibility comparison was performed to characterize this phenomenon, using a well-established non-human primate model, which uses whole, flowing blood in the absence of anticoagulants (Cutiongco et al., 2015; Jurney et al., 2018). This study compared the plasma treated PVA grafts with different microtopographies, untreated PVA grafts (with and without microtopographies), and ePTFE graft controls. Studies have previously revealed that UP PVA have a relatively low platelet accumulation compared to untreated ePTFE, the gold-standard for clinical artificial material vascular grafts (Cutiongco et al., 2015; Jurney et al., 2018; Anderson et al., 2019). As measured in the shunt model, the platelet and fibrin accumulations on the CCLP PVA grafts were significantly higher than the UP PVA grafts, whereas, the 2 μGP PVA grafts did not significantly alter platelet and fibrin accumulations. This was consistent with a previously presented lactate dehydrogenase (LDH) assay absorbance experiment that demonstrated that CCL topographies present the highest platelet activation compared to other topographies (Cutiongco et al., 2015). The CCL topography is, thus, consistently found to be a more favorable surface for platelet activation. The platelet and fibrin accumulation of the other PVA groups were lower than the collagen coated ePTFE positive control and were lower than the ePTFE clinical control grafts. A significant increase in thrombosis was also seen with fresh plasma treatments; however, aged plasma treatment did not significantly increase platelet or fibrin attachment. It is possible that the grafted nitrogen species functional groups, seen with the XPS data even at long time points, encourages platelet and cell adhesion, but the change in surface functional group composition over time during storage in ambient conditions could be reflected in the lower platelet activation on the aged plasma PVA samples (day 44–60) compared to the fresh plasma samples (day 7–11) and enhanced due to exposure of the grafts to the whole blood. Hence, in terms of hemocompatibility, the luminal NH_3_ plasma treatment done between 7 and 11 days prior to graft exposure to whole blood worked well with both UPP and 2 μGP PVA, but not with CCLP PVA. The CCLP PVA had lower platelet and fibrin accumulations after the longer storage time in ambient conditions, and they were comparable to the UPP PVA. It is possible that an ideal storage range of plasma-treated PVA exists which could promote endothelialization *in vivo* without the rapid, initial thrombosis reaction, representing a promising goal for off-the-shelf vascular graft technologies that would encourage long-term clinical patency.

## Conclusions

Research in plasma treatment methods has mainly focused on treating flat surfaces, and there remains a lack of studies investigating luminal plasma treatment of hydrogel-based tubular grafts. Herein, the luminal plasma treatment of small diameter PVA vascular grafts has been reported and characterized. The treatment demonstrated homogeneous luminal surface functionalization and improved endothelial cell attachment. Moreover, the grafted surface functional groups and graft endothelialization were stable up to 30 days, which proved the robustness of luminal surface functionalization. This study proved that plasma treating a tubular shape hydrogel can be done and the treatment was stable even after the hydrogel was swollen. The luminal plasma treatment technique used in this study is therefore a viable option for biomedical applications that require surface modification of tube-like structures such as vascular grafts, nerve conduits, or catheters. The NH_3_-plasma treated unpatterned (UPP) and microgratings (2 μGP) surfaces were shown to not significantly invoke platelet or fibrin activation while the treatment improved PVA grafts endothelialization. Thus, this treatment technique has the potential to improve PVA vascular grafts performance and patency.

## Data Availability

The datasets generated for this study are available on request to the corresponding author.

## Ethics Statement

This study was carried out in accordance with the recommendations of Guide to the Care and Use of Laboratory Animals, Oregon National Primate Research Center (ONPRC) Institutional Animal Care and Use Committee. The protocol was approved by the ONPRC Institutional Animal Care and Use Committee (IP00000300).

## Author Contributions

EY, MTH, DM, and GP were responsible for conception and design of the study. GP, PC, DA, YY, and JT performed data collection. GP, PC, DA, and EY were responsible for data analysis and interpretation. GP and MWH were responsible for statistical analyses. GP was mainly responsible for writing the manuscript. All authors approve this final version of the article.

### Conflict of Interest Statement

The authors declare that the research was conducted in the absence of any commercial or financial relationships that could be construed as a potential conflict of interest.
