# Supplementary material for: Luminal Plasma Treatment for Small Diameter Polyvinyl Alcohol Tubular Scaffolds
Source: Front Bioeng Biotechnol. 2019 May 22;7:117. doi: 10.3389/fbioe.2019.00117 (PMC6541113; doi:10.3389/fbioe.2019.00117)
Supplement: Supplementary file 1 [file Data_Sheet_1.PDF]

Supplementary Material

Luminal Plasma Treatment for Small Diameter Polyvinyl Alcohol Tubular Scaffolds

Grace Pohan, Pascale Chevallier, Deirdre Anderson, John Tse, Yuan Yao, Matthew W. Hagen, Diego Mantovani, Monica T. Hinds, Evelyn K.F. Yim*

*** Correspondence:**
Evelyn K.F. Yim
eyim@uwaterloo.ca

1 Supplementary Figures

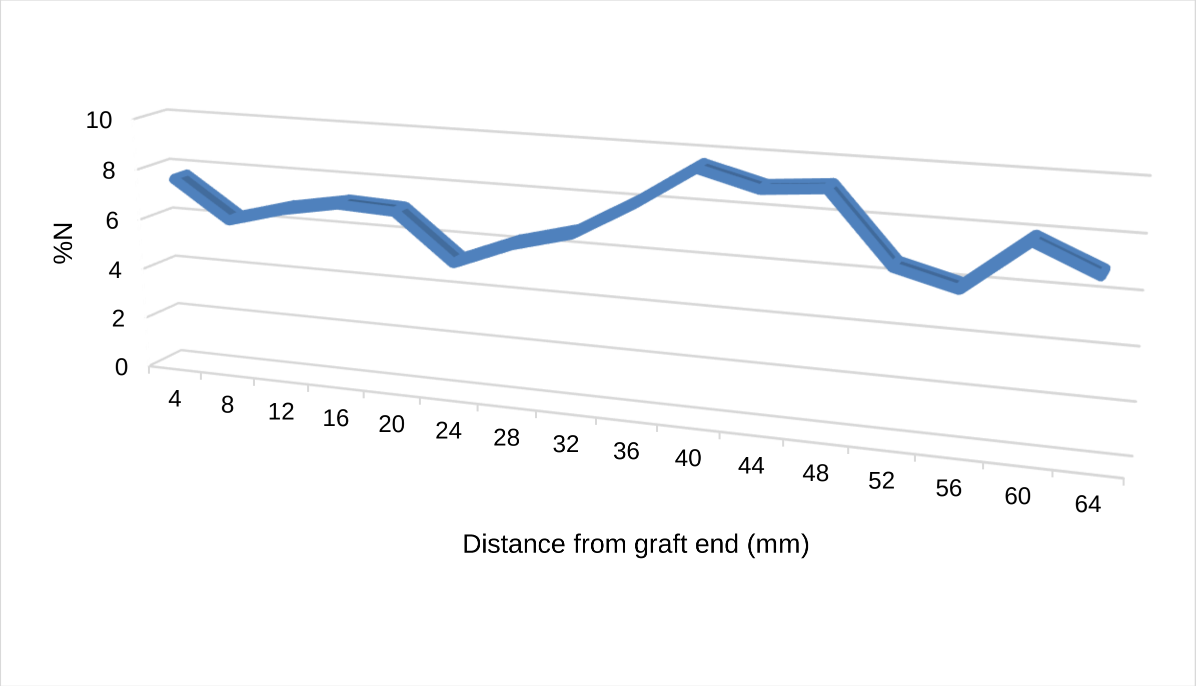

Supplementary Figure 1. Atomic percentage of N measured with low resolution XPS along a 11-cm dehydrated PVA graft. X-axis indicates distance from the graft end.

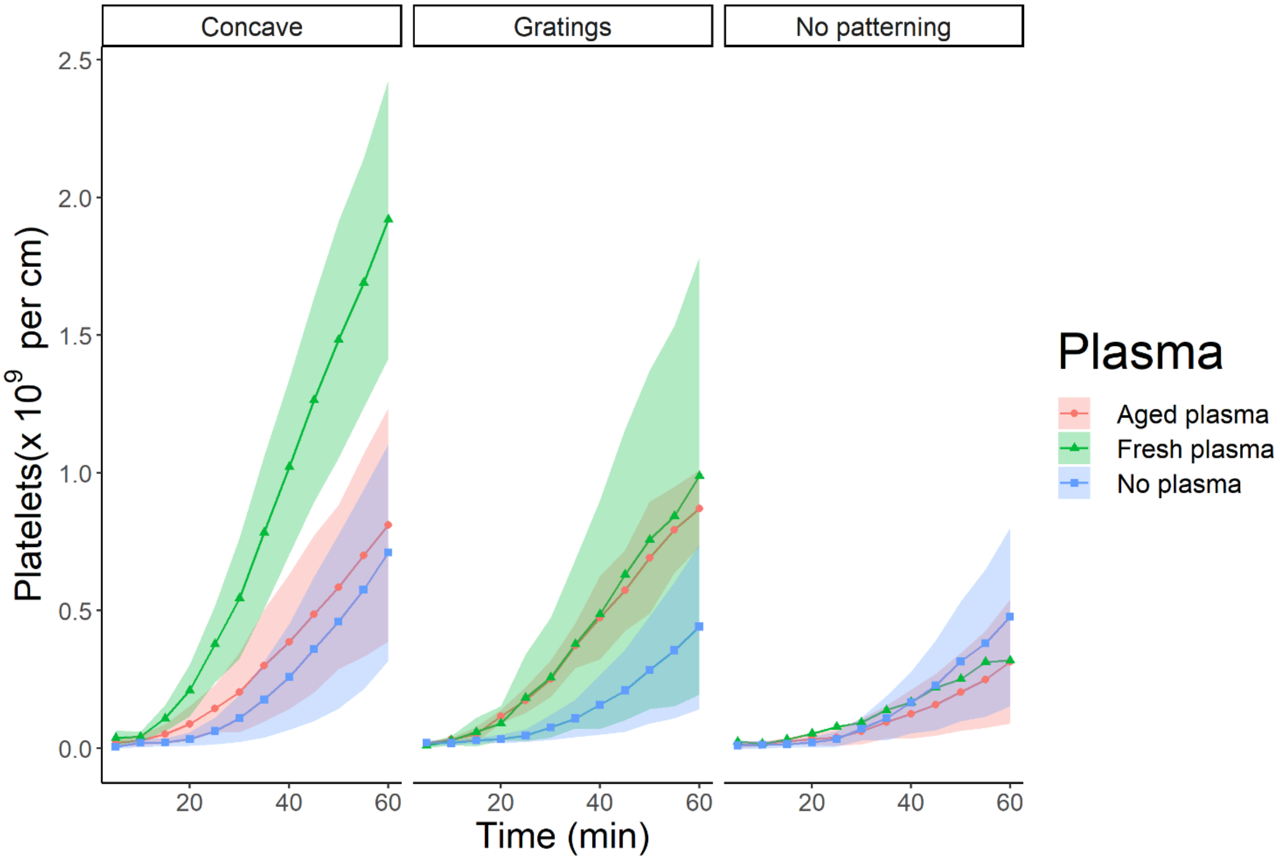

Supplementary Figure 2. Differences in platelet accumulation on patterned and unpatterned PVA graft surfaces between fresh (day 7-11 post-plasma) and aged (day 44-60 post-plasma) plasma samples. Fresh plasma showed a significant increase in platelet attachment compared to no plasma samples (p=0.006); however, this was not reflected when the plasma treatment was aged (p=0.195).

2 Supplementary Table

Supplementary Table 1. XPS results for individual graft sample measured at multiple points along the graft.

	**Untreated**	**Day 0**	**Day 30**	**Day 77**
%C	69.2 ± 1.9%<br>(11 measured pts)	56.4 ± 2.6%<br>(11 measured pts)	59.7 ± 4.7%<br>(10 measured pts)	59.5 ± 1.0%<br>(3 measured pts)
	69.4 ± 1.9%<br>(12 measured pts)	53.6 ± 3.1%<br>(11 measured pts)		
	68.1 ± 1.4%<br>(12 measured pts)	58.8 ± 3.0%<br>(12 measured pts)		
%O	28.1 ± 1.5%<br>(11 measured pts)	29.1 ± 1.9%<br>(11 measured pts)	28.3 ± 2.8%<br>(10 measured pts)	32.1 ± 0.8%<br>(3 measured pts)
	29.4 ± 1.6%<br>(12 measured pts)	31.7 ± 2.0%<br>(11 measured pts)		
	30.2 ± 1.9%<br>(12 measured pts)	23.3 ± 2.0%<br>(12 measured pts)		
%N	0.0 ± 0.0%<br>(11 measured pts)	9.3 ± 1.2%<br>(11 measured pts)	8.4 ± 2.9%<br>(10 measured pts)	5.3 ± 0.3%<br>(3 measured pts)
	0.0 ± 0.0%<br>(12 measured pts)	7.5 ± 2.7%<br>(11 measured pts)		
	0.0 ± 0.0%<br>(12 measured pts)	11.5 ± 1.8%<br>(12 measured pts)		
O/C	0.406 ± 0.032<br>(11 measured pts)	0.518 ± 0.052<br>(11 measured pts)	0.480 ± 0.090<br>(10 measured pts)	0.539 ± 0.021<br>(3 measured pts)
	0.425 ± 0.034<br>(12 measured pts)	0.595 ± 0.060<br>(11 measured pts)		
	0.443 ± 0.036<br>(12 measured pts)	0.398 ± 0.045<br>(12 measured pts)		
N/C	0.000 ± 0.000<br>(11 measured pts)	0.165 ± 0.023<br>(11 measured pts)	0.142 ± 0.050<br>(10 measured pts)	0.088 ± 0.003<br>(3 measured pts)
	0.000 ± 0.000<br>(12 measured pts)	0.142 ± 0.055<br>(11 measured pts)		
	0.000 ± 0.000<br>(12 measured pts)	0.196 ± 0.030<br>(12 measured pts)		

% Peak at 285 eV	43.68 ± 3.15% (11 measured pts)	31.16 ± 4.72% (11 measured pts)	32.85 ± 2.75% (10 measured pts)	34.60 ± 0.43% (3 measured pts)
	37.64 ± 4.35% (12 measured pts)	30.43 ± 3.32% (12 measured pts)		
% Peak at 286.5 eV	23.05 ± 3.33% (11 measured pts)	19.42 ± 4.99% (11 measured pts)	20.01 ± 3.73% (10 measured pts)	19.95 ± 1.07% (3 measured pts)
	28.27 ± 4.24% (12 measured pts)	18.91 ± 3.96% (12 measured pts)		
% Peak at 288.5 eV	2.48 ± 0.86% (11 measured pts)	5.78 ± 1.03% (11 measured pts)	6.89 ± 1.32% (10 measured pts)	4.96 ± 0.85% (3 measured pts)
	2.16 ± 1.01% (12 measured pts)	4.24 ± 1.21% (12 measured pts)		

Supplementary Table 2. Cell number quantification per cm^2 of the *in vitro* cell adhesion study

		**Unpatterned**					**2μm gratings**	**1.8μm concave lenses**
	Day	2	9	12	14	30	30	30
EA.hy926 (cells/cm^2)	Without plasma	4077 (n=1)	3433 (n=1)	9655 ± 6823 (n=2)	5550 (n=1)	256438 (n=1)	48232 (n=1)	70855 (n=1)
EA.hy926 (cells/cm^2)	NH3 plasma	4741 ± 1171 (n=2)	36460 ± 25933 (n=2)	135170 ± 47087 (n=3)	84333 ± 38761 (n=2)	238244 (n=1)	157685 (n=1)	322007 (n=1)
	Day	-	-	-	-	16	16	16
HUVEC (cells/cm^2)	Without plasma	-	-	-	-	11020 ± 996 (n=2)	7680 ± 2325 (n=2)	10728 ± 3862 (n=2)
HUVEC (cells/cm^2)	NH3 plasma	-	-	-	-	28545 ± 20718 (n=2)	90758 ± 18323 (n=2)	41066 ± 38492 (n=2)
